# Anticancer effects and underlying mechanism of Colchicine on human gastric cancer cell lines *in vitro* and *in vivo*

**DOI:** 10.1042/BSR20181802

**Published:** 2019-01-15

**Authors:** Tao Zhang, Wei Chen, Xumian Jiang, Lei Liu, Kai Wei, Hansong Du, Hui Wang, Juan Li

**Affiliations:** Department of Gastrointestinal Surgery, The Center Hospital of Wuhan, TongJi Medical College, HuaZhong University of Science and Techonolgoy, Wuhan 430060, China

**Keywords:** apoptosis, Colchicine, caspase-3, gastric cancer, mitochondrial apoptotic pathways

## Abstract

The present study investigated the effects of Colchicine on gastric carcinoma (GC) cells and explored its possible mechanisms underlying such effects. The results of MTT and colony formation assays showed that Colchicine (2, 5, and 10 ng/ml) markedly inhibited the proliferation of AGS and NCI-N87 cells in a dose-dependent manner. It also led to a reduction in cell migration in both GC cells as determined by Transwell migration assay. Mover, data form Hoechst 33342 staining and flow cytometry assay indicated that Colchicine (2, 5, and 10 ng/ml) promoted the apoptosis of NCI-N87 cells. In addition, the release of cytochrome *c*, the activation of bax, and the inhibition of bcl-2 were observed in NCI-N87 cells treated with Colchicine. Furthermore, the *in vivo* experiment further confirmed that Colchicine administration remarkably suppressed the tumor growth in nude mice via induction of apoptosis at 0.05 and 0.1 mg/kg. In addition, no visible toxicity was observed in liver and renal tissue of mice. This finding suggests that Colchicine-induced apoptosis is associated with caspase-3-mediated mitochondrial apoptotic pathways.

## Introduction

Gastric cancer (GC), the second leading cause of cancer-related deaths worldwide, remains the most frequent type of health issue to human health [1,2]. Surgical resection, chemotherapy, and radiotherapy are currently available therapeutic strategies for GC patients [2]. Chemotherapy is the most effective therapeutic approach for GC patients with unresectable disease by suppressing tumor invasion and metastasis [3], but the results are modest with the overall 5-year survival rates ranging from 5 to 15% [4]. Particularly, severe adverse effects and dose-limiting toxicities of chemotherapy treatments are common [5]. In addition, more than 50% of resected GC patients experience recurrence, metastases, and unavoidable resistance to chemoradiation therapy [6]. Therefore, seeking novel therapeutic agents that reduce the mortality of patients with GC and lower side effects are highly urgent and required. Nowadays, more and more naturally bioactive compounds that can interfere with the essential steps of cancer development have drawn great attention as possible anticancer agents by researchers because of their high activity and low cytotoxicity [7].

Colchicine is an alkaloid of the plant *Colchicum automnale*, and has been used as an anti-inflammatory agent to ameliorate acute gout attacks and other inflammatory disorders for centuries [8]. While, latter studies suggested that Colchicine does not only have notable curative effect on severe hepatitis, post-hepatitic cirrhosis, biliary cirrhosis, and alcoholic cirrhosis, but also has remarkable effect in cancer treatment, such as breast carcinoma, cervical cancer, esophageal cancer, lung cancer, GC, and chronic granulocytic leukemia [9]. Although the antitumor effect of Colchicine is not fully understood, it is widely believed that the activation of apoptosis is probably one reason. In a valuable report, microarray and quantitative reverse transcriptase PCR experiments identified that only up-regulated *DUSP1* gene may contribute to the antiproliferative effects of Colchicine on GC cells and a visible suppressing effect on GC xenograft growth was also observed in nude mice following Colchicine administration [10]. Nevertheless, to the best of our knowledge, their possible anticancer molecular mechanism for GC has remained elusive. The aim of the present study was to investigate the apoptosis-inducing effects of Colchicine in GC cells *in vitro* and *in vivo*, as well as the underlying mechanism. This work would shed light on discovering a safe, natural, and non-toxic agents for achieving anti-GC clinically.

## Materials and methods

### Materials and chemicals

**Figure 1:**
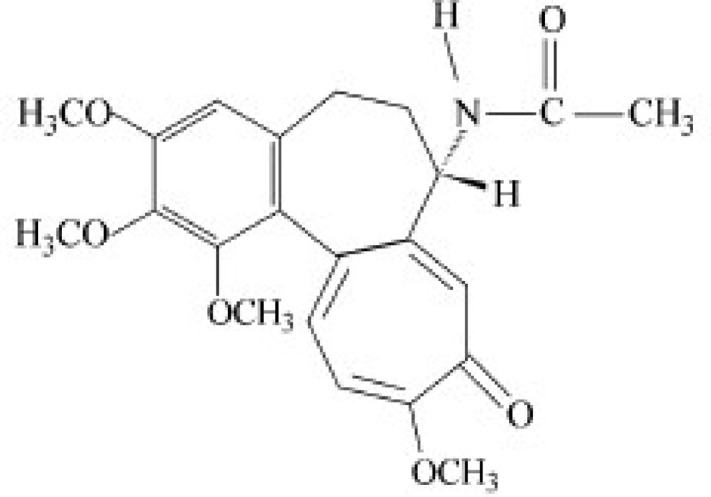
The structure of Colchicine

Purified Colchicine (99.89%, Figure 1), MTT, and DMSO was purchased from Sigma–Aldrich Co. (St. Louis, MO, U.S.A.). Hoechst 33342 and Annexin V-FITC/PI Apoptosis Detection Kit were obtained from KeyGen Biotech (Nanjing, China). Antibodies for bax, bcl-2, cleaved caspase-3, β-actin, and horseradish peroxidase (HRP)–conjugated goat anti-rat secondary antibodies were obtained from Santa Cruz Biotechnology (Santa Cruz Biotechnology, Inc., Dallas, TX, U.S.A.). Rosewell Park Memorial Institute (RPMI) 1640 medium, FCS, antibiotics and FBS were supplied by GE Healthcare Life Sciences (Logan, UT, U.S.A.). All other chemicals were of analytical grade.

### Cell lines and cell culture

The GC cell lines AGS and NCI-N87 was obtained from the Shanghai Institute of Cellular Biology (Chinese Academy of Sciences, Shanghai, China). AGS cells were cultured in Ham’s F12 medium and NCI-N87 cells were routinely maintained in the RPMI-1640 medium in a humidified atmosphere with 5% CO_2_ at 37°C. Both culture mediums consisted of 10% FCS, 5 g/l glucose, 10 mM l-glutamine, 2.0 g/l sodium bicarbonate, 1 mM sodium pyruvate, 100 units/ml penicillin, and 100 μg/ml streptomycin.

### Cell proliferation assay

The effect of Colchicine on cell viability of human GC cells was measured using the MTT assay and colony formation test. For MTT assay, the cells (1 × 10^4^ cells/well) were seeded into 96-well plates overnight followed by treatment with vehicle control (DMSO) or various concentrations of Colchicine (0, 2, 5, and 10 ng/ml) for 48 h. Then, each well was supplemented with 15 μl MTT reagent (5 mg/ml) for 4 h at 37°C in 5% CO_2_. The medium was subsequently removed carefully and 150 μl DMSO was added to each well to dissolve formed formazan crystals. The optical density of each well was read at a wavelength of 490 nm using a microplate reader (Bio-Rad, Nebraska, U.S.A.). The inhibition rate was used to evaluate the cell viability and its algorithm was the following formula: (OD_control_ − OD_treatment_)/OD_control_ × 100% [11]. OD490 in control cells was taken as 100% viability.

For colony formation test, 5 ml of complete medium containing 800 cells was plated into six-well plates and cultured with various concentrations of Colchicine for 24 h. The fresh medium containing Colchicine or not was changed every 3 days. After treatment of 12 days, the supernatant was thrown away, and the cells were then fixed with 5% paraformaldehyde at 4°C for 15 min prior to being stained with 0.5% Crystal Violet solution for 30 min. Finally, the stained colonies were manually counted under an inverted microscope (Nikon, Tokyo, Japan). Cell viability was assayed as relative colony formation rate (%) = number of colonies in the treatment group/number of colonies in the control group × 100%. Assays were carried out in triplicate on three independent experiments.

### Cell migration assay

Cells at 1 ×10^5^ cells/well in 300 μl of serum-free medium were seeded into the upper chamber of a 24-well Transwell insert system (BD Biosciences, San Jose, CA, U.S.A.) with an 8-μm pore size polyethylene terephthalate membrane and 600 μl of 10% FBS-containing medium as a chemoattractant was added to the lower chambers. After 24-h incubation at 37°C in a 5% CO_2_ incubator, non-migrated cells were removed from the upper side of each insert with cotton swab, while the migrated cells on the bottom surface of the insert were carefully washed with PBS, fixed in methanol for 10 min, and then stained with 0.5% Crystal Violet for 20 min. Migrated cells were counted with an inverted microscope at a magnification of ×200 in ten random fields in each well and migration rate was expressed as the percentage of migrated cells compared with that of the control group, which was set as 100%. Each experiment was performed in triplicate.

### Hoechst 33342 fluorescence staining

Nuclear Hoechst 33342 staining was performed to visualize the changes of nuclear morphology of cancer cells after Colchicine treatment. Briefly, at the end of the treatment, cells were collected, washed once with ice-cold PBS, fixed in PBS containing 4% paraformaldehyde for 20 min, and rinsed once with ice-cold PBS. Thereafter, the cells were stained with 10 μg/ml Hoechst 33342 (Hoechst Staining Kit, Beyotime, China) at room temperature in the dark for 30 min, washed twice with PBS, and observed via fluorescence microscopy (Olympus, BX53, Japan). The ratio of apoptotic cells to total cell number was calculated.

### Flow cytometric analysis of apoptosis

The percentage of cells undergoing apoptosis was quantitated with an Annexin V-FITC apoptosis detection kit (BD Bioscience, CA, U.S.A.) according to the manufacturer’s instructions. Briefly, following Colchicine incubation for 24 h, the cells were washed twice with cold PBS, mixed with binding buffer (200 μl) at a concentration of 1 × 10^6^ cells/ml, and added with 5 μl of annexin V-FITC and 5 μl of PI in the dark at room temperature for 5 min. At the end of the incubation period, the cells were then transferred into a flow cytometry tube and analyzed on an FACScan flow cytometer (Becton & Dickinson Co., U.S.A.). The data (10000 events/sample) were collected and analyzed using CellQuest software (Becton Dickinson U.S.A.).

### Western blot analysis

Western blot analysis was conducted as described previously, with slight modification [24]. Briefly, treated or untreated cancer cells were harvested, washed twice with cold PBS, and lysed in Radio Immunoprecipitation Assay (RIPA) buffer (1 mM MgCl_2_, 10 mM Tris/HCl, pH 7.4, 1% Triton X-100, 0.1% SDS, and 1% NP-40). Cell lysates were centrifuged at 12000 rpm for 10 min at 4°C and the concentration of total proteins was determined using a BCA protein assay kit (Beyotime, Shanghai, China). Subsequently, the equal proteins (20 μg) were separated by 12% SDS/PAGE and electrotransferred to 0.45 μm PVDF membranes. After the membranes were blocked with 5% non-fat dry milk in TBS-Tween (TBS-T) including 0.1% v/v Tween-20, the membrane was incubated with specific primary antibodies for bax, bcl-2, cleaved caspase-3, and β-actin at dilutions of 1:1000 overnight at 4°C, and then washed with TBS-T followed by incubation for 1 h with the respective HRP–conjugated goat anti-rat secondary antibodies (1:2000) in TBS-T. The protein signals were visualized using an ECL kit. To assess protein loading, β-actin expression was used as an internal reference.

### Tumor xenograft experiment in nude mice

Male BALB/c nude mice weighing approximately 20 g (4–6 weeks) were supplied by the Laboratory Animal Center of Wuhan University (Wuhan, China) and kept in the facility with free access to a standard diet and water. Animal experimental protocols approved by the Animal Research Ethics Committee of the Center Hospital of Wuhan. NCI-N87 cancer cells (200 μl, 5 × 10^6^) in log phase growth were injected subcutaneously into the plank of the animals. Seven days after tumor inoculation, 40 mice with visible tumor mass in 6–8 mm diameter were divided into four groups of ten mice each. The control group (Group I) was given normal saline (NS) via gavage administration once a day; the positive group (Group II) was given 10 mg/kg 5-Fu via intraperitoneal injection once a day, Colchicine-low dose (group III) and high dose (group IV) groups received gavage administration of 0.05 and 0.10 mg/kg/day of Colchicine, respectively. During the experiment period, tumor volume (TV) and body weight of nude mice were recorded every 5 days. TV was calculated according to the existing formula: TV (mm^3^) = (a^2^ × b)/2, where a and b are the shortest and longest diameters, respectively [12]. TV measurements were conducted in duplicate and recorded as the mean value of each group. All mice were killed by orbital sinus bleeding on day 30 after treatment, and tumors were dissected and weighed. The extent of liver and renal injury were determined by measuring the levels of alanine aminotransferase (ALT), aspartate aminotransferase (AST), blood urea nitrogen (BUN), and serum creatinine (Cr). Then, tumors from each group were fixed in 10% formalin, embedded in paraffin, and cut into 5-μm serial sections for Hematoxylin–Eosin (H&E) and terminal deoxynucleotidyl transferase-dUTP nickend labeling (TUNEL) staining. *In situ* cell death detection kits (Roche Diagnostics, Branchburg, NJ, U.S.A.) were used to detect the apoptosis of paraffin-embedded tumor sections according to the manufacturer’s instructions. The morphological changes and the levels of apoptosis in the tumor tissues were observed and examined under an Olympus fluorescence microscope (Olympus Corp., Tokyo, Japan).

### Statistical analysis

Data are represented as mean ± S.D. of three independent experiments. Statistically significant differences between two groups were calculated using the *t* test, and multiple groups were analyzed by one-way ANOVA. *P*<0.05 was considered to be significant remarkably.

## Results

### Cytotoxic effects of Colchicine on cell growth and migration

**Figure 2:**
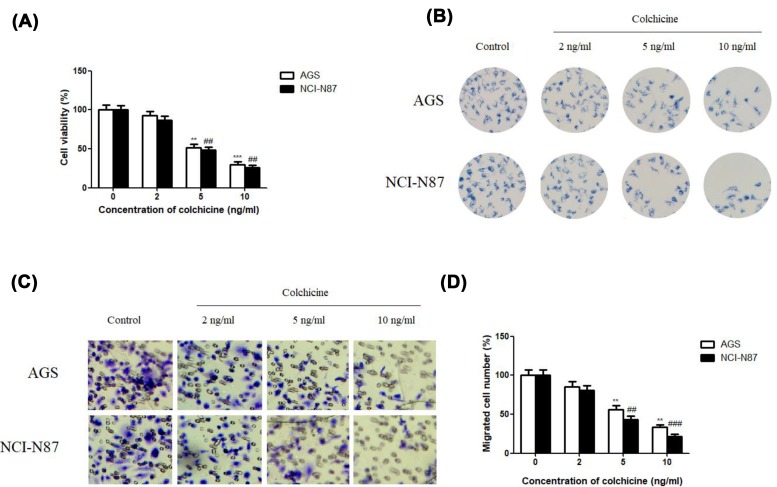
The effect of Colchicine on cell growth and migration (**A**) Effect of Colchicine on the cell viability of human GC AGS and NCI-N87 cells at 48 h. (**B**) Effect of Colchicine on colony formation of human GC AGS and NCI-N87 cells on day 12. (**C**) Effect of Colchicine on the migration of human GC AGS and NCI-N87 cells at 48 h. (**D**) Quantitative analysis of the percentage of migrated cells after treatment with Colchicine at 48 h. Data are means ± S.D. (*n*=5). **P<0.01, ***P<0.001, compared with control of AGS, ^##^*P*<0.01, ^###^*P*<0.001, compared with control of NCI-N87.

First, in the present study, we evaluated the potential cytotoxic effect of Colchicine on the cell growth of AGS and NCI-N87 GC cells using MTT assay. After 48 h of drug exposure, the growth of both cells was significantly inhibited in a dose-dependent manner *in vitro*. Cell viability decreased with the increasing Colchicine concentration, and at each concentration, NCI-N87 GC cells appeared more sensitive to Colchicine than AGS cells (Figure 2A).

Then, we conducted colony formation test to explore the anti-clonogenic effects of Colchicine on both cells. Treatment with Colchicine clearly attenuated the colony-forming ability of AGS and NCI-N87 GC cells, as evidenced by decreased size and number of colonies in Colchicine-treated cells than the control (Figure 2B). What is more, the inhibitory effect of Colchicine on the colony formation of NCI-N87 GC cells was stronger than that in AGS cells. Therefore, these results revealed that Colchicine had a strong cytostatic and cytotoxic effect toward GC cells, especially for NCI-N87 GC cells.

We also carried out a Transwell migration assay to investigate whether Colchicine potentially inhibits GC cell migration. Figure 2C revealed that the treatment of Colchicine dose-dependently inhibited the migration of AGS and NCI-N87 cells (by 85.37% in AGS cells and 80.48% in NCI-N87 cells at a concentration of 2 ng/ml; by 56.35% in AGS cells and 43.42% in NCI-N87 cells at concentration of 5 ng/ml; by 33.15% in AGS cells and 21.7% in NCI-N87 cells at concentration of 10 ng/ml). Interestingly, the number of migrated NCI-N87 cells was less than AGS cells after Colchicine treatment at each same concentration (Figure 2D), although it was not statistically significant (*P*>0.05). Collectively, these results indicated that Colchicine could suppress GC cells proliferation and migration. In the following assay, NCI-N87 GC cells would be chosen as a cell model to make an in-depth research.

### Apoptosis induced by Colchicine

**Figure 3:**
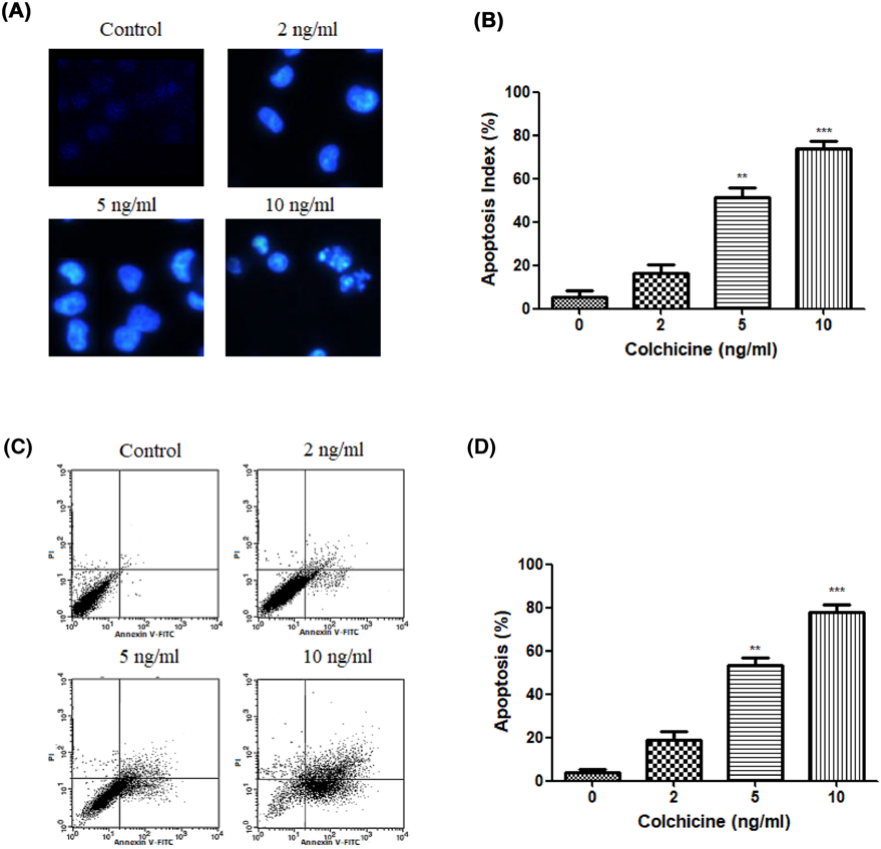
The effect of Colchicine on cell apoptosis (**A**) Morphological analysis of the nuclei of NCI-N87 cells stained by Hoechst 33342 after various concentrations of Colchicine treatment for 48 h (200×). (**B**) Quantitative analysis of the percentage of apoptotic NCI-N87 cells stained by Hoechst 33342 after various concentrations of Colchicine treatment for 48 h. (**C**) Flow cytometry analysis of NCI-N87 cells after various concentrations of Colchicine treatment for 48 h. (**D**) The percentage of apoptotic cells in each group after various concentrations of Colchicine treatment for 48 h. Data are means ± S.D. (*n*=5). **P<0.01, ***P<0.001, compared with untreated control.

Next, flow cytometric analysis and Hoechst 33342 staining assay was employed to reveal whether Colchicine induced-NCI-N87 cell death is mediated through the induction of apoptosis. Hoechst 33258 staining assay in Figure 3A,B indicated that stained NCI-N87 cells following exposure to 2, 5, and 10 ng/ml of Colchicine for 48 h showed a dose-dependent increased number of cells with morphological changes of characteristic apoptosis, including chromatin condensation, bright-phase nuclear fragmentation, and chromosomal DNA fragmentation, whereas untreated NCI-N87 cells displayed a typical image of normal and round intact nuclei.

To further confirm the induction of apoptosis in NCI-N87 cells by Colchicine treatment, we also investigated the proportion of apoptotic cells on a flow cytometry by Annexin V-FITC/PI dual-labeling technique. As shown in Figure 3C,D, the percentage of apoptotic cells was increased greatly by Colchicine to 18.54, 54.34, and 76.6%, at 2, 5, and 10 ng/ml, respectively.

**Figure 4:**
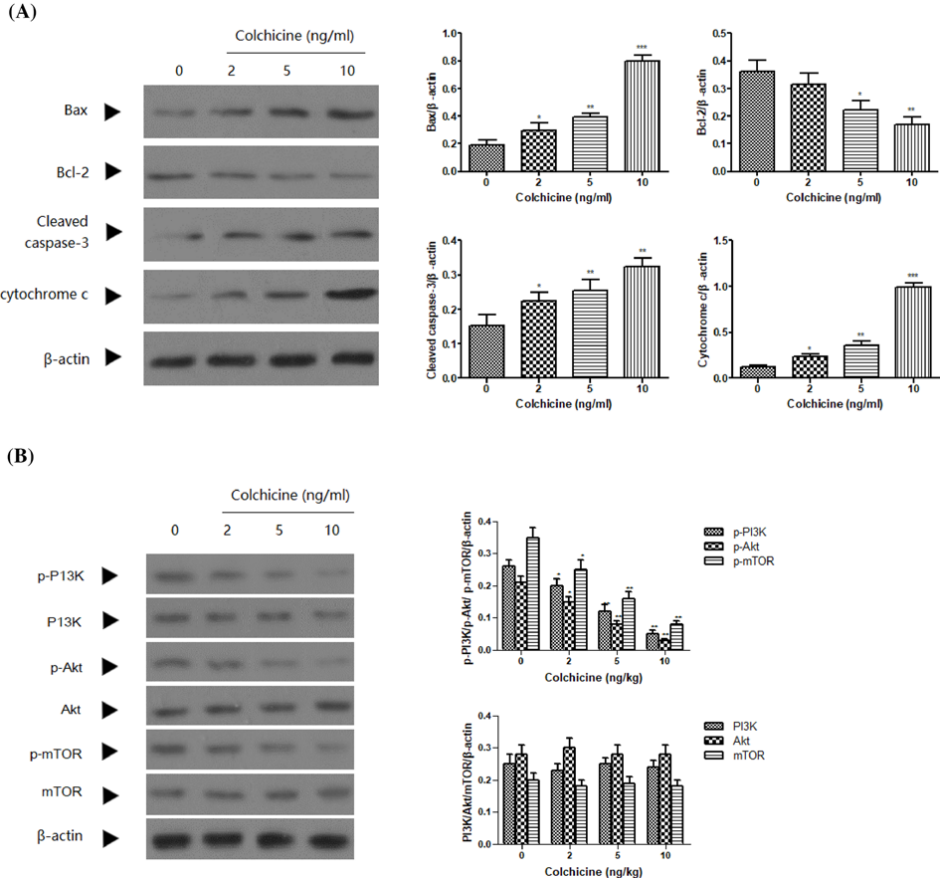
The effect of Colchicine on apoptotic protein expression (**A**) The protein expression of bax, bcl-2, cleaved-caspase-3, and cytochrome *c* in NCI-N87 cells after various concentrations of Colchicine treatment for 48 h. (**B**) The protein expression of PI3K, p-PI3K, Akt, p-Akt, mTOR, and p-mTOR in NCI-N87 cells after various concentrations of Colchicine treatment for 48 h. Data are means ± S.D. (*n*=5). *P<0.05, **P<0.01, ***P<0.001, compared with untreated control.

To further delineate the mechanism by which Colchicine induced apoptosis in GC cells, Western blot assay was conducted to detect apoptosis-suppressed protein in NCI-N87 cells, including cleaved caspase-3, Bax, Bcl-2, cytochrome *c*, PI3K, p-PI3K, Akt, p-Akt, mTOR, and p-mTOR. As shown in Figure 4A, treatment with Colchicine for 48 h significantly increased the expression of cleaved caspase-3 and pro-apoptotic protein (Bax), decreased apoptosis-suppressed protein (Bcl-2), and significantly promoted the release of cytochrome *c* from the mitochondria to the cytoplasm (*P*<0.05, 0.01, or 0.001) compared with the control. Most importantly, treatment with Colchicine showed no effect on the protein levels of PI3K, Akt, and mTOR, but significantly decreased the levels of p-PI3K, Akt, and mTOR in NCI-N87 cells when compared with the control cells (Figure 4B, *P*<0.05 or 0.01).

All the above data indicated that Colchicine treatment induced caspase-3-dependent apoptosis in NCI-N87 cells via suppressing the PI3K/Akt/mTOR signaling pathway.

### Effect of Colchicine on tumor development *in vivo*

**Figure 5:**
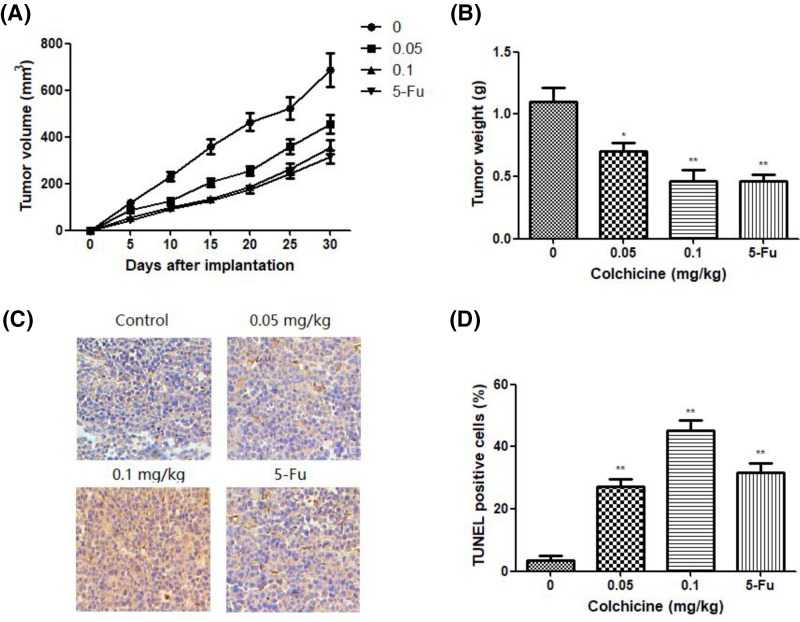
The effect of Colchicine on tumor growth and apoptosis in vivo (**A**) The antitumor effects of Colchicine on TV of xenograft model of NCI-N87 cells. (**B**) The antitumor effects of Colchicine on tumor weight of xenograft model of NCI-N87 cells. (**C**) Apoptotic cells were detected in xenograft tumor tissue using the TUNEL assay (200×). (**D**) Quantitated results of the TUNEL assay. Data are means ± S.D. (*n*=10). *P<0.05, **P<0.01, compared with untreated control.

Further, the effect of Colchicine on the growth of GC cells *in vivo* was examined in a xenograft model established in BALB/c nude mice following subcutaneous injection of NCI-N87 cells. TV was recorded every 5 days and the TV was clearly suppressed in mice treated with 0.05 or 0.10 mg/kg/day of Colchicine at days 5, 10, 15, 20, and 25 compared with the corresponding values in the control group treated with NS (Figure 5A). After treatment, the mean tumor weights in different groups were also measured, and results showed that the tumor weight in the colchicine-treated mice was significantly lower than in the control mice (*P*<0.05, Figure 5B). The tendency of TV inhibition in different groups was consistent with tumor weight inhibition.

We next examined tumor apoptosis induced by Colchicine *in vivo* by TUNEL staining. As shown in Figure 5C,D, the apoptotic index of the Colchicine-treatment group was significantly high than that from the control group, as determined by counting the number of positively TUNEL-labeled cells in random microscopic fields (*P*<0.05).

### Evaluation of side effects

At the end of the experiment, no death or sign of toxicity was observed in all experimental nude mice. ALT, AST, BUN, and Cr levels in the serum were measured to evaluate the effect of Colchicine on the function of liver or renal. No significant variations of four parameters in Colchicine-treated mice were observed compared with control mice (*P*>0.05, Table 1), indicating no injury to liver or renal organs. In addition, there was no significant change in body weight during the treatment period between the Colchicine-treated mice and the control mice (data not shown).

**Table 1:** The effect of Colchicine on the function of liver or renal of mice

Group	*n*	ALT (U/l)	AST (U/l)	Urea (μmol/l)	Cr (μmol/l)
Group I	10	25.36 ± 3.12	110.21 ± 12.21	4.54 ± 0.56	13.22 ± 1.82
Group II	10	24.12 ± 2.87	114.55 ± 12.04	5.01 ± 0.62	14.12 ± 1.54
Group III	10	23.58 ± 2.65	113.87 ± 11.76	4.88 ± 0.57	13.82 ± 1.65
Group IV	10	24.11 ± 2.41	114.02 ± 11.64	4.71 ± 0.55	13.74 ± 1.57

## Discussion

The results of *in vitro* study from MTT assay and colony formation assay showed that the treatment of Colchicine could effectively inhibit cell viability and migration of both AGS and NCI-N87 cells in a concentration-dependent manner compared with untreated cells. Similarly, the inhibitory effect on cell migration by Colchicine was also observed on both cell lines as determined by Transwell migration assay. These results indicated that Colchicine may exhibit a potential therapeutic effect on GC. Notably, NCI-N87 cell line has a higher sensitivity to Colchicine treatment than AGS cells, characterized with a lower cell viability and a higher migration ability, but the differences between them at each concentration are not significant (*P*>0.05). To gain deep mechanistic insights into the cytotoxic activity of Colchicine on GC cells, we further use only the more sensitive cell line NCI-N87 in the next experiment.

Apoptosis induction by natural product-derived anticancer agents is a critical tactic to eliminate cancer cells [13,14] and the ability to induce apoptosis in cancer cells is a key criterion for screening anticancer drugs [15]; as such, the inhibition of GC cell viability and migration following Colchicine treatment may result from apoptosis. In this regard, to ascertain whether the apoptosis contributed to the cytotoxicity of Colchicine on NCI-N87 cells, Hoechst 33342 staining was employed to reveal the morphological changes in the apoptotic cells. The apoptotic morphological characteristics, such as reduction in cellular volume, bright nuclear condensation or fragments, were observed in NCI-N87 cells following 2, 5, and 10 ng/ml of Colchicine treatment, while untreated control cells displayed normal and round nuclei. Further, flow cytometry was performed to detect the proportion of apoptotic cells with Annexin V and PI staining. Following treatment with 0, 2, 5, and 10 ng/ml of Colchicine, the dose-dependent apoptosis rate was increased greatly from 3.54 to 76.6%. The similar apoptosis induction effect of Colchicine was also evaluated in breast cancer MCF-7 cells by Sun et al. [16]. These results suggest that Colchicine effectively induced apoptosis in NCI-N87 cells.

It is well accepted that apoptosis is a type of programmed cell death organized by a series of signal cascades to maintain tissue and cell hemostasis [17]. However, if this programmed cell apoptosis is disrupted in cancer cells, it causes overgrowth of malignant cells [18]. During the development of apoptosis, the anti-apoptotic bcl-2 protein mainly distributed in the mitochondrial membrane and the cytoplasm can stabilize the mitochondrial membrane and block cytochrome *c* release, while pro-apoptotic bax protein translocated to the outer membrane of mitochondria, causing the release of cytochrome *c*, and finally resulting in the activation of caspase-9 and caspase-3, which serves one of the key apoptosis executors that trigger the final process of apoptosis [19,20]. The imbalance between bax and bcl-2 has been recognized as an index to modulate the process of apoptosis [21]. Western blot analysis provide the evidence that treatment with Colchicine inhibited the expression levels of bcl-2 proteins, but increased that of bax, cytochrome *c*, and caspase-3 to promote cell apoptosis. These changes in apoptotic proteins were also observed in human colon cancer HT-29 and human normal liver L-02 cells following Colchicine treatment [22,23].

The PI3K/Akt/mTOR signaling pathway plays an important role in the regulation of cell cycle, proliferation, survival, angiogenesis, and autophagy [24,25]. There is accumulating evidence that the PI3K/AKT/mTOR pathway is frequently activated in diverse cancer and drugs that target the PI3K/Akt/mTOR signaling pathway have the potential to induce apoptosis in cancer cells [26,27]. The present Western blotting results showed that the phosphorylation of PI3K, AKT, and mTOR was suppressed in NCI-N87 cells following exposure to Colchicine. However the expression of PI3K, Akt, and mTOR were not affected. These data collectively suggested Colchicine induced caspase-3-mediated apoptosis via suppressing the PI3K/Akt/mTOR signaling pathway in NCI-N87 cells.

In addition, *in vivo* antitumor study demonstrated that administration of Colchicine (0.05 and 0.1 mg/kg) could effectively inhibit the TV and tumor weight. TUNEL assays of the subcutaneous tumor tissues from each group demonstrated that Colchicine has the same apoptosis induction activity in the tumor mass as *in vitro*, while almost no apoptosis was observed in the control group. More importantly, ALT, AST, BUN, and Cr levels in the serum revealed that no toxicity of Colchicine on liver or renal tissue, indicating it is safe under the current dosage.

## Conclusion

Taken together, these findings provide evidence and shed new light on the application of Colchicine as a novel potential candidate for GC treatment. However, the underlying molecular mechanisms for antitumor activities of Colchicine still need to be further investigated.

## Abbreviations

ALT: alanine aminotransferase
AST: aspartate aminotransferase
BUN: blood urea nitrogen
Cr: creatinine
GC: gastric cancer
HRP: horseradish peroxidase
NS: normal saline
RPMI: Rosewell Park Memorial Institute
TBS-T: TBS-Tween
TUNEL: terminal deoxynucleotidyl transferase-dUTP nickend labeling
TV: tumor volume
